# Context-Specific Osteogenic Potential of Mesenchymal Stem Cells

**DOI:** 10.3390/biomedicines9060673

**Published:** 2021-06-12

**Authors:** Aleksandra Kostina, Arseniy Lobov, Daria Semenova, Artem Kiselev, Polina Klausen, Anna Malashicheva

**Affiliations:** Laboratory of Regenerative Biomedicine, Institute of Cytology Russian Academy of Science, 194064 Saint Petersburg, Russia; aleksandrakostina1991@gmail.com (A.K.); arseniylobov@gmail.com (A.L.); daria.semenova1994@gmail.com (D.S.); artem.kiselyov@gmail.com (A.K.); polina.klauzen@gmail.com (P.K.)

**Keywords:** Notch, osteogenic differentiation, mesenchymal stem cells, aortic valve interstitial cells, calcification

## Abstract

Despite the great progress in the field of bone tissue regeneration, the early initiating mechanisms of osteogenic differentiation are not well understood. Cells capable of osteogenic transformation vary from mesenchymal stem cells of various origins to mural cells of vessels. The mechanisms of pathological calcification are thought to be similar to those of bone formation. Notch signaling has been shown to play an important role in osteogenic differentiation, as well as in pathological calcification. Nevertheless, despite its known tissue- and context-specificity, the information about its role in the osteogenic differentiation of different cells is still limited. We compared mesenchymal stem cells from adipogenic tissue (MSCs) and interstitial cells from the aortic valve (VICs) by their ability to undergo Notch-dependent osteogenic differentiation. We showed differences between the two types of cells in their ability to activate the expression of proosteogenic genes *RUNX2*, *BMP2*, *BMP4*, *DLX2*, *BGLAP*, *SPRY*, *IBSP*, and *SPP1* in response to Notch activation. Untargeted metabolomic profiling also confirms differences between MSCs and VICs in their osteogenic state. Analysis of the activity of *RUNX2* and *SPP1* promoters shows fine-tuned dose-dependency in response to Notch induction and suggests a direct link between the level of Notch activation, and the proostogenic gene expression and corresponding osteogenic induction. Our data suggest that osteogenic differentiation is a context-dependent process and the outcome of it could be cell-type dependent.

## 1. Introduction

Mesenchymal stem cells of various origins (MSCs) have high proliferative and differentiation potentials, but the mechanisms and factors affecting the ability of cells to transform into tissue-specific somatic cells remain largely unclear [1]. A comprehensive understanding of the mechanisms of MSCs differentiation, as well as the ability to control and regulate the process of differentiation, are parts of both fundamental and applied problems. The identification of early triggers of osteogenic differentiation is important for a wide range of biomedical applications, from enhancing this type of differentiation in order to restore bone tissue, to the suppression of ectopic osteogenic differentiation. For example, vascular calcification is a serious complication, the initiating mechanisms of which are not completely understood, but it is well accepted that the pathological vascular calcification resembles bone formation [2,3,4]. At the same time, recent data suggest that stem cells of various origins could be different in their osteogenic responsiveness and potential [5].

The MSCs of adipogenic tissue are very broadly used to study various differentiation mechanisms and to develop regenerative protocols including osteogenic differentiation [1]. Aortic valve interstitial cells (VICs) are a well-known model for pathological calcification [6].

Notch signaling plays an important role in skeletal development and bone remodeling [7,8]. Controversial data exist regarding the role of Notch activation in promoting or preventing osteogenic differentiation [7,9,10,11,12,13,14,15], but it is well accepted that Notch signaling is critical for the formation of bone tissues [7,8]. We have recently shown that the activation of Notch promotes the osteogenic differentiation of adipose-tissue derived MSCs in a dose-dependent manner, but high dosage of Notch activation could have a negative influence and prevent osteogenic differentiation of MSCs [16]. At the same time, dysregulation of Notch signaling is associated with pathological calcification of the aortic valve and pathological osteogenic transformation of valve interstitial cells (VICs) in patients with calcific aortic valve disease [17]. Notch also contributes to the osteogenic transformation of aortic smooth muscle cells [18].

Thus, we assumed that there are might be differences in the effect of Notch-signaling on the osteogenic differentiation of mesenchymal cells from various tissues. Here, we sought to compare mesenchymal stem cells of adipose tissue and aortic valve interstitial cells in terms of their ability to undergo Notch-dependent osteogenic differentiation.

## 2. Results

### 2.1. MSCs of Adipose Tissue are More Sensitive to Notch-Dependent Induction of Osteogenic Differentiation in Comparison to VICs

**Figure 1:**
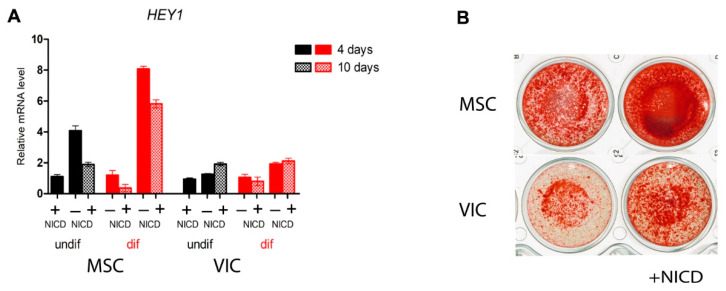
Mesenchymal stem cells (MSCs) of adipose tissue are more sensitive to the Notch-dependent induction of osteogenic differentiation in comparison with aortic valve interstitial cells (VICs). The cells were transduced with the lentiviral Notch intracellular domain (NICD) and osteogenic differentiation was induced. The expression of Notch target *HEY1* was analyzed by qPCR after either 4 or 10 days of differentiation (**A**) and osteogenic differentiation was analyzed by alizarin staining (**B**).

We analyzed the ability of the Notch signaling pathway to influence osteogenic differentiation of MSCs and VICs. Notch was activated by lentiviral transduction of the Notch intracellular domain (NICD). In addition to NICD transduction, we induced osteogenic differentiation by an osteogenic medium (Figure 1). The MSCs turned out to be more sensitive than VICs to the induction of osteogenic differentiation both in the absence and in the presence of Notch activation (Figure 1). Nevertheless, the activation of Notch enhanced the osteogenic differentiation of both cells.

### 2.2. Proosteogenic Gene Expression Is Different between MSCs and VICs at Osteogenic Differentiation

**Figure 2:**
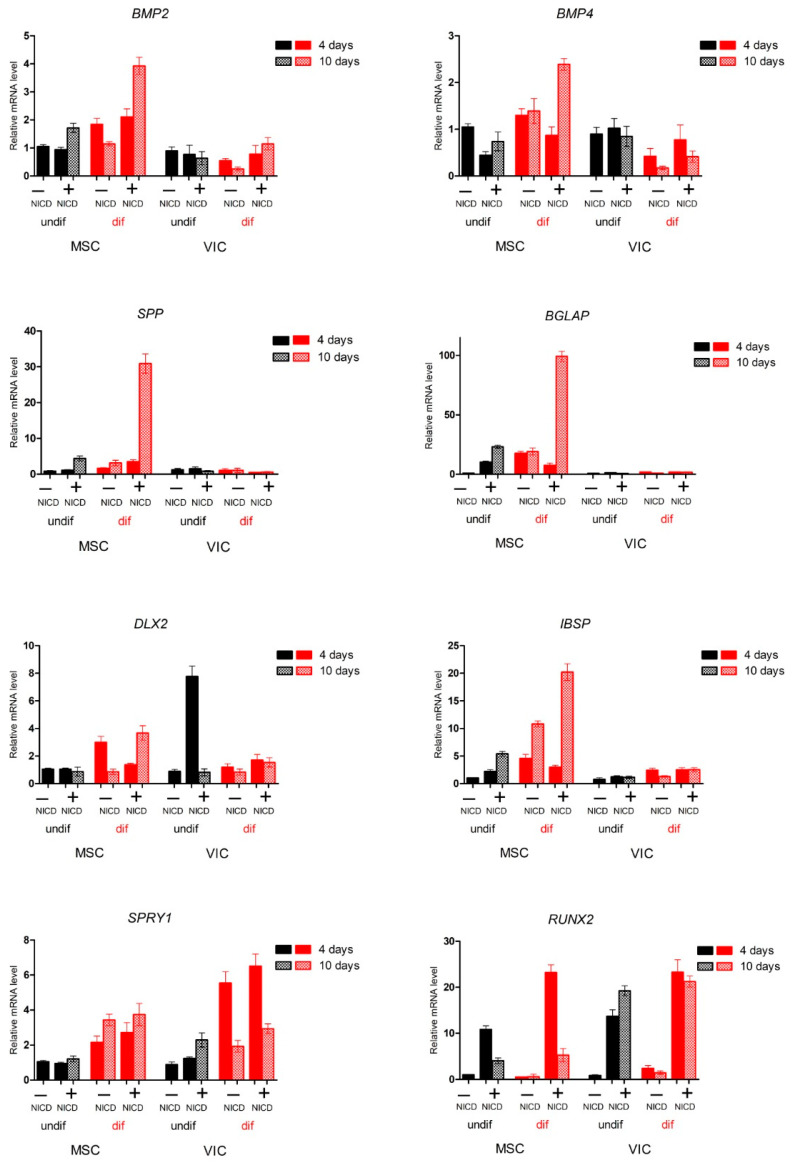
Mesenchymal stem cells (MSCs) of adipose tissue are different in their sensitivity to the Notch-dependent induction of osteogenic differentiation in comparison with aortic valve interstitial cells (VICs). The cells were transduced with the lentiviral Notch intracellular domain (NICD) and osteogenic differentiation was induced. The expression proosteogenic genes was analyzed by qPCR after either 4 or 10 days of differentiation.

Next, we analyzed the proosteogenic gene expression in the MSCs and VICs after the osteogenic induction and activation of Notch (Figure 2). Analysis of the expression of a panel of proosteogenic markers revealed a number of markers: Secreted Phosphoprotein 1 (SPP1, also known as Osteopontin), Bone Gamma-Carboxyglutamate Protein (BGLAP), Integrin Binding Sialoprotein (IBSP), and Bone Morphogenetic Protein 2/4 (BMP2/4) that were activated only in MSCs in response to the induction of osteogenic differentiation. The expression of proosteogenic markers was enhanced in the presence of Notch activation. The expression of the key proosteogenic marker Runt-Related Transcription Factor 2 (RUNX2) significantly increased in the presence of Notch activation upon the induction of osteogenic differentiation, and decreased in MSCs by day 10 of osteogenic differentiation compared with VICs.

### 2.3. Metabolic Profiles of MSCs and VICs Differ after Osteogenic Differentiation

To assess the overall physiological proximity of VICs and MSCs after osteogenic differentiation, we performed untargeted metabolomic profiling of ethanol extracts of these cells by GC–MS. Metabolite extraction was combined with following Alizarin Red stain (Figure 1).

**Figure 3:**
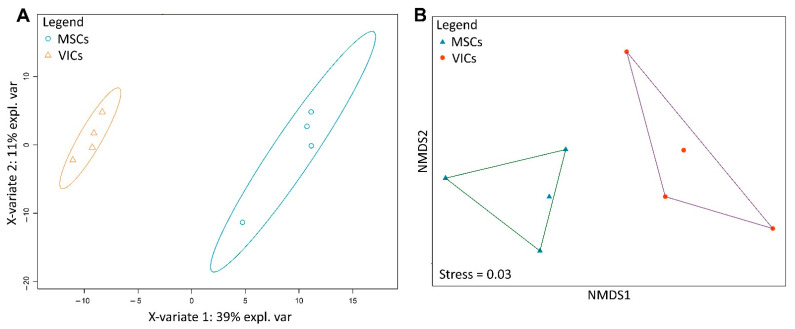
Ordination plots illustrating the differences in the metabolomic profiles among mesenchymal stem cells (MSCs) of adipose tissue and aortic valve interstitial cells VICs after osteogenic differentiation induced by a standard osteogenic medium or osteogenic medium, and transduction with Notch intracellular domain (NICD). (A) Partial least squares discriminant analysis (PLS-DA) scores plot. (**B**) Non-metric multidimensional scaling (nMDS) plot. The Kruskal stress value indicating the quality of ordination.

We identified 277 metabolomic features and performed ordination based on their intensity using two statistical methods: classification by partial least squares discriminant analysis (PLS-DA) and ordination by non-metric multidimensional scaling (nMDS). Both methods demonstrate similar results. The MSCs and VICs formed two distinct clusters not only in classification (Figure 3a), but also in the ordination (Figure 3b). Thus, the physiological differences of these cell types did not disappear during osteogenic differentiation—VICs and MSCs differentiate to physiologically different osteoblast-like cells.

### 2.4. Assessment of the Transcriptional Activity of the Promoters of the Proosteogenic Markers RUNX2 and SPP1 Depending on the Level of Notch Activation

Notch signaling is known to be extremely dose-dependent and cell-lineage specific [19]. RUNX2 is a key factor in the regulation of osteogenic processes. The induction of osteogenic differentiation in cell cultures causes an increase in the expression of the transcription factor RUNX2 (Figure 2). In view of the fact that the Notch signaling pathway enhances the osteogenic differentiation of mesenchymal cells, we set a task to determine whether the intracellular activation domain of the Notch1 receptor directly regulates the activity of the *RUNX2* promoter, as well as another marker of osteogenic differentiation, *SPP1*.

**Figure 4:**
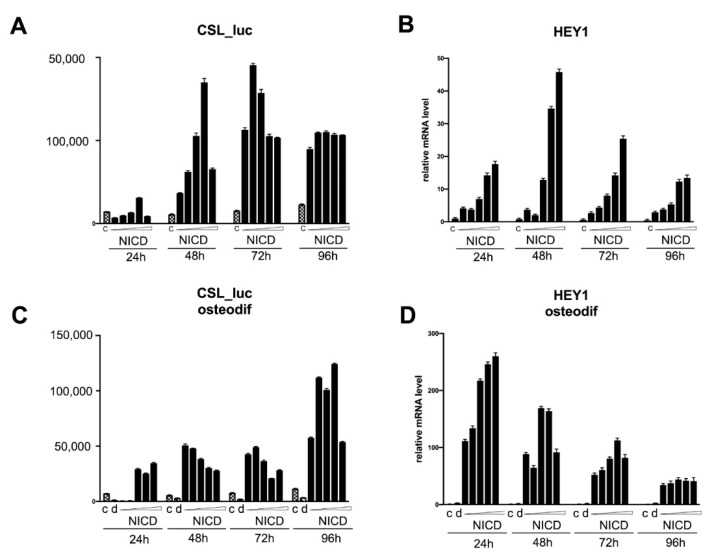
Analysis of the dose-dependent Notch activation influence on CSL promoter activity (**A**, **C**) and *HEY1* transcription (**B**,**D**) in mesenchymal stem cells (MSCs) of adipose tissue in non-osteogenic (**A**,**B**) and osteogenic conditions (**C**,**D**). The cells were transduced with the construct bearing CSL-luciferase reporter and subsequently with various doses of NICD-bearing viruses, and were analyzed in non-osteogenic and osteogenic conditions after indicated periods of time.

**Figure 5:**
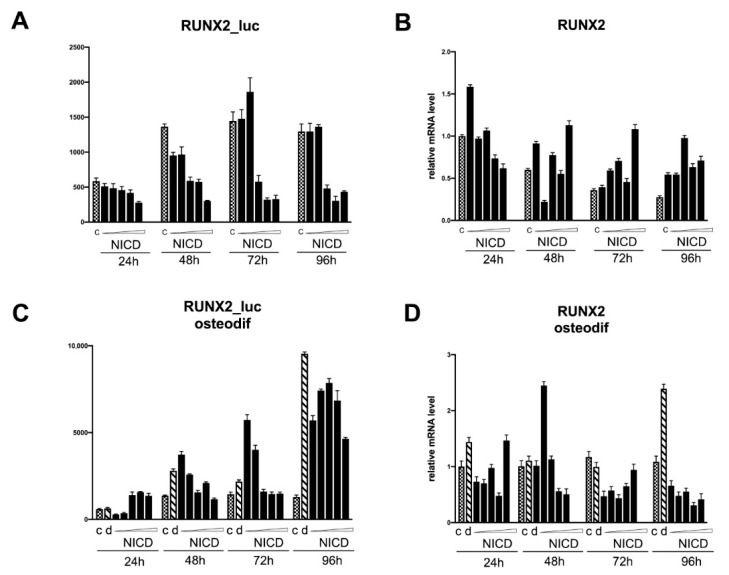
Analysis of the dose-dependent Notch activation influence on *RUNX2* promoter activity (**A**,**C**) and *RUNX2* transcription (**B**,**D**) in mesenchymal stem cells (MSCs) of adipose tissue in non-osteogenic (**A**,**B**) and osteogenic conditions (**C**,**D**). The cells were transduced with the construct bearing CSL-luciferase reporter and subsequently with various doses of NICD-bearing viruses, and were analyzed in non-osteogenic and osteogenic conditions after indicated periods of time.

**Figure 6:**
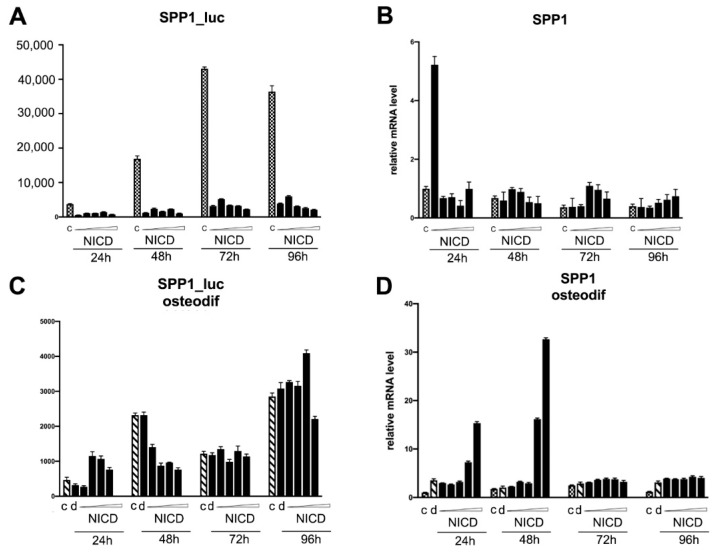
Analysis of dose-dependent Notch activation influence on SPP1 promoter activity (**A**, **C**) and SPP1 transcription (**B**,**D**) in mesenchymal stem cells (MSCs) of adipose tissue in non-osteogenic (**A**,**B**) and osteogenic conditions (**C**,**D**). The cells were transduced with the construct bearing CSL-luciferase reporters and subsequently with various doses of NICD-bearing viruses, and were analyzed in non-osteogenic and osteogenic conditions after indicated periods of time.

We transduced MSCs with reporter lentiviral constructs, in which the luciferase gene was set under the control of either *RUNX2* or *SPP1* promoters. We dosed the activation of the signaling by introducing different doses of NICD-bearing lentiviruses. Subsequently, osteogenic differentiation was induced in the cells. The cells transduced with a reporter bearing CSL-responsive element (main co-activator of Notch-dependent transcription complex) were used to control the dose-dependent Notch activation. The analysis of the activity of promoters was carried out by assessing the expression of luciferase at 24, 48, 72, and 96 h after the induction of osteogenic differentiation, as well as in non-osteogenic conditions (Figure 4). In parallel with the analysis of the luciferase activity, we assessed the level of mRNA expression of the Hairy/enhancer-of-split related with YRPW motif protein 1 (*HEY1*), *SPP1*, and *RUNX2* genes (Figure 4, Figure 5 and Figure 6, correspondingly). As expected, dose-dependent NICD activation of Notch signaling under non-osteogenic conditions resulted in a dose-dependent increase in 12xCSL activity with a maximum transcriptional activity at 48 and 72 h (Figure 4A,C). At the same time, we observed a drop in 12xCSL activity at high doses of NICD, at the highest dose at 48 h, and at medium and higher doses at 72 h. By 96 h, the dose-dependent difference in the activity of 12xCSL had disappeared. The dynamics of the transcriptional activity of 12xCSL under osteogenic conditions was different (Figure 4A,C). We observed a noticeable increase in activity already at 24 h at doses of NICD of above average, at 48 h we noted the maximum level of activity at the lowest dose of NICD, followed by a drop in activity. At 96 h of analysis, we observed significantly higher absolute values of the luciferase level compared with the same time period under non-osteogenic conditions, along with an earlier increase in 12xCSL activity under osteogenic conditions. We suggest that this indicates an increased sensitivity of cells to Notch signals under osteogenic conditions. The expression of the *HEY1* gene confirmed the data obtained from the analysis of the transcriptional activity of 12xCSL. Under non-osteogenic conditions, the *HEY1* expression increased according to the dose of NICD with the highest expression level at 48 and 72 h, but with no decrease in the expression at high doses (Figure 4B,D). Under osteogenic conditions, the values of the relative level of *HEY1* expression were significantly higher compared with the non-osteogenic conditions. The highest expression level was observed already at 24 h, followed by a decrease in time and alignment with the NICD dose. The data on the *HEY1* mRNA expression support the assumption that cells are more sensitive to Notch signals under osteogenic conditions.

The transcriptional activity of the RUNX2 promoter upon the activation of Notch (Figure 5) did not differ from the control level in non-osteogenic conditions; at doses above average, a decrease in the basal level of promoter activity was observed (Figure 5, A). The expression level of RUNX2 mRNA in non-osteogenic conditions also did not differ from the control without Notch activation (Figure 5B). Under osteogenic conditions, there was a regular increase in promoter activity compared with cells under non-osteogenic conditions (Figure 5C). Notch activation increased the activity of the RUNX2 promoter at 24 h at high doses, and at 48 and 72 h at the minimum dose of NICD, with a drop in the activity level to the control level at high doses of NICD. The expression level of RUNX2 mRNA was markedly increased in cells with osteogenic differentiation by only 96 h and did not differ in cells with Notch activation (Figure 5D).

The level of activity of the *SPP1* promoter was quite high in the cells without Notch activation under non-osteogenic conditions, and strongly decreased in the cells with Notch activation (Figure 6). Under osteogenic conditions, we did not observe a significant increase in the activity of the SPP1 promoter neither in cells without Notch activation, nor in cells with activated Notch (Figure 6A,C). The level of expression of *SPP1* mRNA under osteogenic conditions and Notch activation dose-dependently increased at 24 and 48 h, which is in favor of a possible positive regulation of *SPP1* expression by the Notch signaling pathway, but not through direct regulation of the activity of the promoter of this gene (Figure 6B,D). The role of the osteomarker SPP1 in osteogenic processes requires further study.

The data obtained on the transcriptional activity of 12xCSL Notch elements and promoters of key osteomarkers, in particular RUNX2, indicate, on the one hand, a possible positive role of Notch in the early stages of osteogenic processes, and, on the other hand, the dependence of these stages on the fine tuning of the dose and duration of Notch activation.

## 3. Discussion

Our findings suggest that the fine-tuned dosage of Notch signaling is critical for the osteogenic differentiation of mesenchymal stem cells of different origins. The cells have different sensitivities to proosteogenic stimuli and to Notch activation.

We suggest that the initial level of the Notch signal could be critical for the cell fate decision in relation to osteogenic differentiation. By altering the amount of ligands and receptors expressed in a cell, numerous scenarios of Notch activation patterns can be generated [20]. We recently showed the lineage-specific differences in Notch receptors and ligands expression among the cells of a similar mesenchymal origin [21]. The obtained comparative data on the tissue specificity of the expression of Notch components indicate that the individual level and ratio of expression of different Notch components can influence the differentiation decisions of cells. In general, this concept is consistent with the idea of a very finely tuned system of the entire Notch apparatus, when even a small shift in the expression of one of the components can shift the differentiation fate of the cell. How this tight regulation is controlled at a molecular level is the subject of future studies.

Notch-signaling is extremely dose sensitive because of the lack of a signal amplification step or the utilization of secondary messengers to transmit the signal from the cell surface to the nucleus [22]. Our data on the Notch-dependent activation of the *RUNX2* promoter suggests that the regulation of the promoter could be directly connected to Notch-dependent transcription. This could potentially explain the extreme sensitivity of osteogenic differentiation to Notch activation. However, the exact mechanism of interaction between Notch signaling and the *RUNX2* gene requires further research.

Our metabolite analysis suggested that VICs and MSCs differentiated to physiologically different osteoblast-like cells. The extraction of metabolites was combined with the Alizarin red stain, so we could directly compare the calcium level and physiological similarity of VICs and MSCs. We demonstrated that a combination of classic Alizarin Red stain routinely used for the confirmation of osteogenic differentiation with metabolomic profiling is fruitful. This combination might be widely used for the assessment of the physiological similarity of the investigated cells.

The data from several research groups indicate that differences exist between various types of mesenchymal stem cells in their ability to osteogenic differentiation. Human MSCs derived from bone marrow, synovial fluid, adult dental pulp, and exfoliated deciduous teeth showed distinct characteristics for the osteogenic, chondrogenic, adipogenic, and neurogenic differentiation potentials [23].

For example, canine MSCs harvested from different sources showed a distinct osteogenic potential. Comparative proteomics-based systems biology analysis was used to study the osteogenic differentiation behavior by MSCs harvested from bone marrow and dental pulp. Dental pulp stem cells contained superior osteogenicity compared with bone marrow-derived MSCs. Global analyses by the proteomics data showed distinct protein clustering and expression patterns upon the *in vitro* osteogenic induction between them [5].

In spite of many controversial data, several lines of evidence directly suggest active participation of Notch in osteogenic differentiation. Thus, Jagged1 activates Notch signaling and subsequently promotes osteogenic differentiation in human periodontal ligament cells [24]. Both human dental pulp cells and human periodontal ligament cells expressed higher Notch target gene (Hairy and Enhancer of Split-1 (HES1) and HEY1) when cells were seeded on a Jagged1 immobilization surface [25]. The gene expression profiling of osteogenic differentiation of human bone marrow-derived mesenchymal stromal cells in vitro and the bone healing period of the murine tibial fracture in vivo shows that various Notch signaling components are differentially expressed during osteogenic differentiation of human bone marrow-derived mesenchymal stromal cells *in vitro* and bone healing period of murine tibial fracture *in vivo* [26]. It has been shown recently that canonical Notch signaling is required for bone morphogenetic protein-mediated human osteoblast differentiation [27,28].

It is obvious that the activation of Notch signaling orchestrates the progression of mesenchymal progenitor cells through the osteoblast lineage, but there is a limited understanding of the ligand- and receptor-specific functions. Paracrine Notch signaling through non-osteoblastic cell types contributes additional layers of complexity and requires further research.

## 4. Materials and Methods

### 4.1. Isolation, In Vitro Culture, and Differentiation of Primary Cell Lines

The study was carried out using primary human mesenchymal stem cells from adipose tissue (MSCs) [29] and aortic valve interstitial cells (VICs) from the aortic valves of patients with calcific aortic valve disease [16,17]. Primary cells were cultured for 3–6 passages. MSCs were maintained in tissue culture grade uncoated Petri dishes (Corning Incorporated, Corning, NY, USA) in alpha-MEM medium (PanEco, Moscow, Russia), supplemented with 10% fetal calf serum (Hyclone Laboratories, Logan, UT), 50 units/mL penicillin, and 50 μg/mL streptomycin (Invitrogen, Carlsbad, CA, USA) at 37 °C and 5% CO_2_. VICs were maintained in tissue culture grade Petri dishes (Corning Incorporated, Corning, NY, USA) coated with 0,1 % gelatin solution (Thermo Scientific, Waltham, MA, USA) in a DMEM medium (Thermo Scientific, Waltham, MA, USA) supplemented with 10% fetal calf serum (Hyclone Laboratories, Logan, UTHyclone, USA), 50 units/mL penicillin, and 50 μg/mL streptomycin (Invitrogen, Carlsbad, CA, USA) at 37 °C and 5% CO_2_.

The immunophenotype of the MSCs and VICs was verified with a flow cytometer Guava EasyCyte 8 (Millipore, Burlington, MA, USA) using CD19, CD34, CD45, CD73, CD90, and CD105 monoclonal antibodies (BD Biosciences, San Jose, CA, USA) as previously described [30].

Osteogenic differentiation for both types of cells was induced through the addition of 50 μM ascorbic acid, 0.1 μM dexamethasone, and 10 mM beta-glycerophosphate (Sigma Aldrich, St. Louis, MO, USA) to the culture medium. The culture media was changed twice a week. The differentiation was considered terminal after 21–22 days of induction.

Calcium deposits were demonstrated by Alizarin Red staining. The cells were washed with PBS, fixed in 70% ethanol for 60 min, washed twice with distilled water, and stained using Alizarin Red solution (Sigma Aldrich, St. Louis, MO, USA).

### 4.2. Genetic Constructs and Lentivirus Production

Lentiviral packaging plasmids were a generous gift of Dr. Trono (École Polytechnique Fédérale de Lausanne, Switzerland). Lentiviral production was performed as described previously [21]. In brief, 100-mm dishes of subconfluent 293T cells were co-transfected with 15 μg pLVTHM-T7, 5.27 μg pMD2.G, and 9.73 μg pCMV-dR8.74psPAX2 packaging with a PEI reagent. The following day, the medium was changed to the fresh one, and the cells were incubated for 24 h to obtain a high-titer virus production. The produced lentivirus was concentrated from a supernatant by ultracentrifugation, resuspended in 1% BSA/PBS, and frozen in aliquots at −80 °C. The virus titer was defined by a GFP-expressing virus; the efficiency of the transduction was 85–90% by GFP.

The viruses bearing coding sequencings of the Notch intracellular domain (NICD) were described previously [31].

To generate OPN-luc-LegoIG2 lentiviral plasmid containing luciferase coding sequence under the control of the OPN promoter, as a source of OPN promoter-luciferase construction, we used OPN(-1206)-luc plasmid. OPN(-1206)-luc was a gift from Gerhart Ryffel (Addgene plasmid # 31106; http://n2t.net/addgene:31106, accessed date 11 June 2021; RRID:Addgene_31106). After amplification with HerculaseII DNA polymerase (Agilent Technologies, Santa Clara, CA, USA) and primers containing NheI and NotI restriction enzyme sites, we subcloned OPN-luc construction to the LeGO-iG2 backbone in the place of the SFFV promoter preserving IRES-GFP and a polylinker. LeGO-iG2 was a gift from Boris Fehse (Addgene plasmid # 27341; http://n2t.net/addgene:27341, accessed date 11 June 2021; RRID:Addgene_27341). The promoter region of RUNX2 gene was selected using UCSC browser with the FANTOM5 database, the region of 2000 bp upstream of the RUNX2 start codon of the first exon was chosen as the region most covered by transcription factor binding sites. This region was amplified using HerculaseII DNA polymerase and primers containing NheI and XhoI restriction enzyme sites. This region was cloned in the LeGO-iG2 backbone in the place of the SFFV promoter preserving IRES-GFP and a polylinker.

### 4.3. qPCR Analysis

The total RNA from the cultured cells was isolated using ExtractRNA (Eurogen, Moscow, Russia). RNA (1 μg) was reverse transcribed with a MMLV RT kit (Eurogen, Moscow, Russia). Real-time PCR was performed with 1 μL cDNA and SYBRGreen PCR Mastermix (Eurogen, Moscow, Russia) in the Light Cycler system using specific forward and reverse primers for the target genes. The primer sequences are represented in Table 1. The thermocycling conditions were as follows: 95 °C for 5 min, followed by 45 cycles of 95 °C for 15 s and 60 °C for 1 min. A final heating step of 65 °C to 95 °C was performed to obtain the melting curves of the final PCR products. Changes in the target genes expression levels were calculated as fold differences using the comparative ∆∆CT method. The mRNA levels were normalized to GAPDH mRNA.

### 4.4. Promoter Activity Assay

To estimate the Notch activity, we transfected cells with a lentiviral 12XCSL-Luciferase reporter construct and measured the transactivation of the CSL promotor using luciferase activation. Cell lysis was performed using the Luciferase Assay System (Promega, Madison, WI, USA), according to the manufacturer recommendations. Luciferase activity was measured with Synergy2 (BioTek Instruments, Inc., Winooski, VT, USA). Samples were normalized by protein content using a Pierce BCA Protein Assay Kit (Thermo Scientific, Waltham, MA, USA).

### 4.5. Metabolomic Analysis

Untargeted metabolomic profiling was used to investigate the overall physiological similarity of the MSCs and VICs in osteogenic differentiation. The extraction of metabolites was combined with the Alizarin Red stain: after fixation in 70% ethanol for 1 h, the obtained ethanol extracts were transferred to ultracentrifuge tubes, dried under vacuum at room temperature in CentriVap benchtop vacuum concentrator (Labconco), and stored at -20 °C until use. For metabolomic analysis, the samples were dissolved in 15 μL pyridine (Sigma-Aldrich, St. Louis, MO, USA) and converted to trimethylsilyl derivatives (silylation) by adding 15 μL N,O-bis-(trimethylsilyl)trifluoroacetamide containing 1% trimethylchlorosilane (BSTFA + TMCS; Supleco, Bellefonte, PA, USA) and analyzed by gas chromatography–mass spectrometry in a Pegasus 4D (Leco, St. Joseph, MI, US) system with a HP5-MS capillary column (30 m × 0.25 mm I. D., film thickness of 0.25 µm; Agilent Technologies, Santa Clara, CA, USA). Helium was used as the carrier gas at a constant rate of 1 mL/min. The injector temperature was 320 °C, MS source temperature was 230 °C, and column temperature program consisted of injection at 70 °C with an increase of 4 °C per min to 320 °C, followed by 320 °C for 15 min. The MS was operated in the electron impact mode with an ionization energy of 70 eV. The scan range was set from 50 to 800 Da with an acquisition rate of 10 spectra/s.

Quantification was performed in the MS-DIAL software (Ver. 4.0) [32]. Pyridine was used as an internal standard for the quantification of analytical results. 277 metabolite features were quantified. Obtained quantitative data were analyzed in R (Ver. 3.6.1) [33] by quantile normalization by limma package [34] with further ordination of samples by non-metric multidimensional scaling (nMDS) [35] and partial least square-discriminant analysis (PLS-DA) [36].

## Figures and Tables

**Table 1:** Primers for the human genes used in the study.

Gene	Primer 5′–3′	
*HEY1*	forward	TGGATCACCTGAAAATGCTG
	reverse	CGAAATCCCAAACTCCGATA
*GAPDH*	forward	CAAGGTCATCCATGACAACTTTG
	reverse	GTCCACCACCCTGTTGCTGTAG
*RUNX2*	forward	TGGATCACCTGAAAATGCTG
	reverse	CGAAATCCCAAACTCCGATA
*BGLAP*	forward	CCTCACACTCCTCGCCCTAT
	reverse	CTTGGACACAAAGGCTGCAC
*IBSP*	forward	GATTTCCAGTTCAGGGCAGTAG
	reverse	CCATAGCCCAGTGTTGTAGC
*DLX2*	forward	GGAGCCCCCATCCCTTATCTT
	reverse	ATCCGCAAAGGCACCTAAACT
*SPRY1*	forward	GGACCCAGCCCAAGCAA
	reverse	TTGTGCTGTGTCAGGTCCTCTT
*BMP2*	forward	GCCAAGCCGAGCCAACAC
	reverse	CCCACTCGTTTCTGGTAGTTCTTC
*BMP4*	forward	AGCACTGGTCTTGAGTATCCTG
	reverse	GCAGAGTTTTCACTGGTCCC
*SPP1*	forward	TCACCTGTGCCATACCAGTTAAA
	reverse	TGGGTATTTGTTGTAAAGCTGCTT

## Data Availability

Data available upon request.
